# Characteristic Structural Knowledge for Morphological Identification and Classification in Meso-Scale Simulations Using Principal Component Analysis

**DOI:** 10.3390/polym13162581

**Published:** 2021-08-04

**Authors:** Natthiti Chiangraeng, Michael Armstrong, Kiattikhun Manokruang, Vannajan Sanghiran Lee, Supat Jiranusornkul, Piyarat Nimmanpipug

**Affiliations:** 1 Department of Chemistry, Faculty of Science, Chiang Mai University, Chiang Mai 50200, Thailand; natthiti.c@gmail.com (N.C.); armstrongmichael119@gmail.com (M.A.); kiattikhun.m@cmu.ac.th (K.M.); 2 Department of Chemistry, Faculty of Science, University of Malaya, Kuala Lumpur 50603, Malaysia; vannajan@um.edu.my; 3 Center of Excellence for Innovation in Analytical Science and Technology (I-ANALY-S-T), Chiang Mai University, Chiang Mai 50200, Thailand; 4 Department of Pharmaceutical Sciences, Chiang Mai University, Chiang Mai 50200, Thailand; supat.jira@cmu.ac.th

**Keywords:** polystyrene, polyisoprene, morphology, copolymer, structure factor, PCA

## Abstract

Meso-scale simulations have been widely used to probe aggregation caused by structural formation in macromolecular systems. However, the limitations of the long-length scale, resulting from its simulation box, cause difficulties in terms of morphological identification and insufficient classification. In this study, structural knowledge derived from meso-scale simulations based on parameters from atomistic simulations were analyzed in dissipative particle dynamic (DPD) simulations of PS-*b*-PI diblock copolymers. The radial distribution function and its Fourier-space counterpart or structure factor were proposed using principal component analysis (PCA) as key characteristics for morphological identification and classification. Disorder, discrete clusters, hexagonally packed cylinders, connected clusters, defected lamellae, lamellae and connected cylinders were effectively grouped.

## 1. Introduction

Self-assembly of block copolymers resulting in an order-structural formation in polymeric materials has been extensively studied due to the wide range of potential applications [1,2]. Diblock copolymers comprised of chemically incompatible blocks that consist of only two dissimilar blocks bound together have attracted much attention due to the fact that, despite their simple chemical structures, they can give rise to rich morphological behavior [3,4,5]. Sphere-liked micelles, cylinders, gyroids and lamellae are the characteristically observed morphologies [3,4,5,6].

A target morphology and its potential applications were investigated from an experimental point of view and confirmed using various characterization techniques [7,8,9], including vibrational spectroscopy (IR/Raman), differential scanning calorimetry (DSC), scanning electron microscopy (SEM), transmission electron microscopy (TEM), wide-angle X-ray diffraction (WAXD), small-angle X-ray scattering (SAXS), small-angle neutron scattering (SANS), etc. Complementary results were also utilized to achieve the goal. Weiyu et al. reported that typical morphologies of polyethylene-*block*-polyethylene oxide (PE-*b*-PEO) were successfully identified using the previously mentioned scientific instruments and that the temperature was responsible for their morphological transitions [9]. Morphological changes from lamellae structures to gyroid, hexagonally packed, cylindrical and spherical structures due to temperature were revealed and discussed in detail for the case of PE_17_-*b*-PEO_40_. The authors also reported that the changes in these components during morphological transformations could be varied because of their different intrinsic properties, such as temperature responsiveness and so forth. Khandpur and co-workers used a combination of dynamic mechanical spectroscopy (DMS), TEM, SEM and SANS to indicate and classify morphologies of polystyrene-*block*-polyisoprene (PS-*b*-PI) copolymers with PI volume fractions ranging from 0.24 to 0.82 [3,4]. The study showed that ordinary morphologies can be detected and observed. The phase diagram was constructed to show a possible region in which a particular morphology could be observed. The experimental result was in line with the theoretical study undertaken by Cochran and co-workers [10].

Modeling tools can be used to successfully visualize a morphology obtained from simulations. Nevertheless, it can be problematic to distinguish irregular morphologies with intricate structures in the complex arrangements. Typically, an order parameter is utilized to distinguish the appearance of the simulated morphologies. This function is often implemented in computational routines in order to be useful in material modeling. In dynamic mean-field density functional (DDF) [11,12] theory, a transformation of morphology from the beginning to the end of simulation can be investigated using order parameters defined in terms of the mean squared deviation from homogeneity in volume *V* [6,13]. The order parameter is given by

(1) $$\mathrm{order}\;\mathrm{parameter}=\frac{1}{V}\int_{V}\left[\eta_{i}^{2}\left(r\right){-\;\eta }_{i}^{2}\;\right]\;dr,$$

where $\eta_{i}$ is the dimensionless density for species *i*. The tendency of the order parameters can be used to indicate the transformation of morphologies from one to another as well as to confirm the stable morphology. A high value of the order parameter refers to possible phase segregation, whereas components are more miscible when the value is close to zero [13]. Another coarse-grained simulation is the dissipative particle dynamic (DPD) approach implemented in the DL_MESO software package [14]. An output gives three different eigenvalues over a simulation time. The isosurface normal distribution *p*(**n**) can be constructed using the obtained densities and the average value of the overall density, which is used as the isosurfaces’ threshold. The second moment or symmetric tensor **M** is written as

(2) M=∫nnp(n)dn,

in which **M** describes an ideal of how the particles are distributed and arranged in the system. The solution of this equation provides three eigenvalues $\mu_{i}$, where $\overset{3}{\underset{i=1}{\sum }}\mu_{i}=1$ can be used as the order parameter for morphological identification and classification similarly as described above [15,16]. This order parameter can distinguish a type of morphology into three main mesophases, including isotropic ($\mu_{1}\approx \mu_{2}\approx \mu_{3}$), cylindrical ($\mu_{1}\ll \mu_{2},\;\mu_{3}$) and lamellar ($\mu_{1},\;\mu_{2}\ll \mu_{3}$) mesophases [5]. The criteria used to judge here were proposed by Preinsen et al. [15] and Warren et al. [16] However, the arrangements of some morphologies are too sophisticated to distinguish solely using a visualizer or order parameter, as described in our previous paper [5].

To overcome this limitation, principal component analysis (PCA) is introduced and utilized in this study. PCA is a suitable method because it can reduce dimensionality in the datasets into a few principal components that are explainable and understandable [17]. Each data point can be plotted and can display the cluster of the calculated datasets. The contribution of each classification along with the squared cosine (cos^2^), or the quality of representations, can provide a visual explanation of how important the data are in each classification and how much data are compressed in each component [18]. It can generate a set of new variables, namely principal components (PCs), to represent datasets of interest. This algorithm aims to reduce the dimensionality of a dataset to emphasize the main and important variations in the data. With the PCA algorithm, an original matrix (*X*) of the dataset can be transformed into two new matrices, which is given by

(3) X=TP+E,

where *T* is a score matrix, *P* is a loading matrix and *E* is a residual containing the variation. The score plot provides the location of the samples and the loading plot indicates correlations among variables. PCA has successfully been used to project a mean squared displacement (MSD) in datasets analyzed from simulation trajectories to explain the mechanistic insights of biological molecules [19]. Fernandez and coworkers revealed that the projection of the datasets of the atomic property-weighted radial distribution functions (RDFs) to the first and second principal components can successfully distinguish geometrical properties and gas uptake capacities of metal-organic frameworks [20].

In light of this information, we realized that an improvement to the method for classification of a simulated morphology type was necessary and challenging. The developed method could be a valuable and useful tool for other researchers to identify and classify an observed morphology easily, precisely, and accurately by using PCA. In this study, morphologies of PS-*b*-PI diblock copolymer were explored using DPD simulations. A suitable condition that gives a variety of morphologies was selected from our previous paper [5] as a demonstration. PCA was applied to distinguish the significance of morphologies that are difficult to identify using only physical appearance and order parameters. The sets of structural properties were used as crucial variables in the PCA. The observed quantitative data are useful for the classification of morphologies correctly.

## 2. Methods

### 2.1. Dissipative Particle Dynamics (DPD) Simulation and the Analysis of an Apparent Morphology at an Equilibrium and Its Related Order Parameters

**Figure 1:**
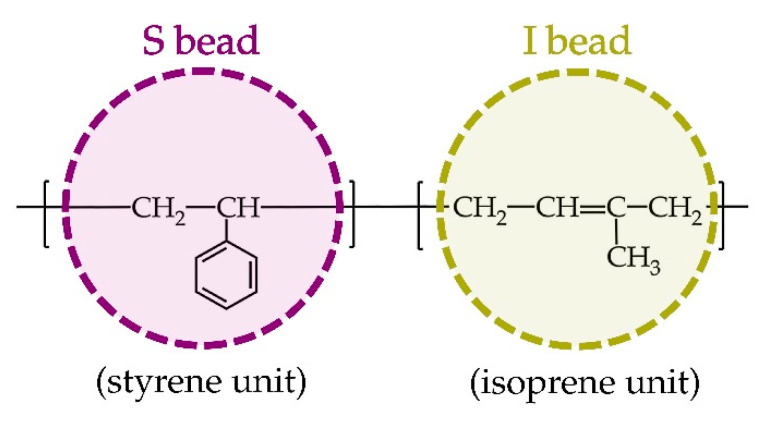
Chemical structures of styrene and isoprene units and their corresponding coarse-grained beads in the simulation system.

Chain assembly of polystyrene-*block*-polyisoprene (PS-*b*-PI) diblock copolymer was explored by means of dissipative particle dynamics simulations. The elementary unit in DPD is a spherical particle representing a fluid element, which in turn represents linearly connected chains composed of S- and I-type DPD particles for styrene and isoprene, respectively. The molecular structures and their corresponding coarse-grained models for both types of DPD particles are illustrated in Figure 1. All boxes contained 24,000 coarse-grained particles with a density σ of 3.0. Each particle was connected by a spring with a constant $C_{ij}$ of 4. The additional parameters are provided in Table S1. The primitive coarse-grained structures reported in reference [5] were used and created using the molecule-generation tool (molecule.exe) in the DL_MESO software (2.7 rev 08, Daresbury Laboratory, Daresbury, UK) package [14]. We selected the coarse-grained models S_1_I_19_, S_2_I_18_, S_3_I_17_, S_4_I_16_, S_5_I_15_, S_6_I_14_, S_7_I_13_, S_8_I_12_, S_9_I_11_, S_10_I_10_, S_11_I_9_, S_12_I_8_, S_13_I_7_, S_14_I_6_, S_15_I_5_, S_16_I_4_, S_17_I_3_, S_18_I_2_ and S_19_I_1_ with a volume fraction range from 0.05 to 0.95. This condition was chosen because of the variety of simulated morphologies observed in a symmetric phase diagram [5] carried out at 393 K, which corresponded well with the experimental observations by Khandpur and co-workers [3,4]. In this DPD simulation, each system was firstly equilibrated for 1,000,000 steps with a step size of 0.01. Subsequently, a production run was carried out until a morphology reached an equilibrium state. All DPD simulations were carried out using the DL_MESO software package.

For data manipulations, we used export_image_vtf.exe to extract the final structure of coarse-grained structures and subsequently used VMD software (1.9.3, Theoretical and Computational Biophysics Group, Urbana, IL, USA) [21] to convert a structure into a pdb format file for visualization. ParaView [22] was used to visualize the structure of the coarse-grained beads, the isodensity of each component with a color gradient and an isosurface showing a surface between the different species in a simulation. The program isosurfaces.exe was utilized to extract a file in the VTK format for visualization of the isodensity and isosurface, which were calculated to the order parameter. The radial distribution function (RDF) and its Fourier-space counterpart, S(*k*), were computed using the embedded rdfmol.exe tool in DL_MESO software. In this study, the last 1,000,000 simulation steps from each model were used for the analysis. The proposed methodology employed entirely free-to-use software available to academic and non-commercial sectors.

### 2.2. Principal Component Analysis (PCA)

In this study, PCA analysis was performed and visualized using R programming language [23]. All calculations were performed using available built-in packages. The RDF and S(*k*) datasets of 19 different coarse-grained models providing 7 distinct morphologies were coded as variables. In this work, a range of radius in DPD units from 0.085 to 9.995 was selected for the set of RDF data while a range of *k* from 0.30 to 315.00 was chosen for the set of S(*k*) data. In the calculation of the PCA, a selected range of RDF and S(*k*) values were separately designated as individual data points for each variable. Before performing the PCA, we checked the distribution of the data points to ensure that there was a major contribution into the first two principal components (PC1 and PC2). Then, we carried out full PCA calculations.

The Factoextra [24] and Corrplot [25] packages were additionally used for visualizing the PCA results. The Factoextra package was used to generate the loading plots and to show a classification of variables of interest. The Corrplot package was used to generate a correlation plot of their contributions and quality values in order to observe the compression of each variable. The size of the circle and the color shade in this plot represent the contribution and quality in each PC, respectively. The eigenvalues of the quality were used to indicate the quality of representations. Using this package, such values are typically normalized to a value of −1 to 1 and represented by a color gradient. The PCA script for structural knowledge analysis and data visualization was provided in Script S1.

## 3. Results

### 3.1. Radial Distribution Function (RDF) and Structure Factor (S(k)) Analysis of Coarse-Grained Structures

In order to investigate the equilibrium morphologies of the PS-*b*-PI copolymer, the copolymeric chains were mapped onto coarse-grained beads by comparing the specific volume of isoprene or styrene to that of water molecules. In our previous study we reported that the isoprene or styrene monomer is approximately equivalent to a single coarse-grained bead [5]. This finding is also consistent with the results reported by Li and coworkers [26]. In this study, 19 coarse-grained models were constructed based on our previous study [5], specifically S_1_I_19_, S_2_I_18_, S_3_I_17_, S_4_I_16_, S_5_I_15_, S_6_I_14_, S_7_I_13_, S_8_I_12_, S_9_I_11_, S_10_I_10_, S_11_I_9_, S_12_I_8_, S_13_I_7_, S_14_I_6_, S_15_I_5_, S_16_I_4_, S_17_I_3_, S_18_I_2_ and S_19_I_1_. After that, their equilibrium morphologies were explored through DPD simulations. From the DPD simulations, the coarse-grained models aggregated into a variety of characteristic morphologies at a simulation temperature of 393 K.

**Figure 2:**
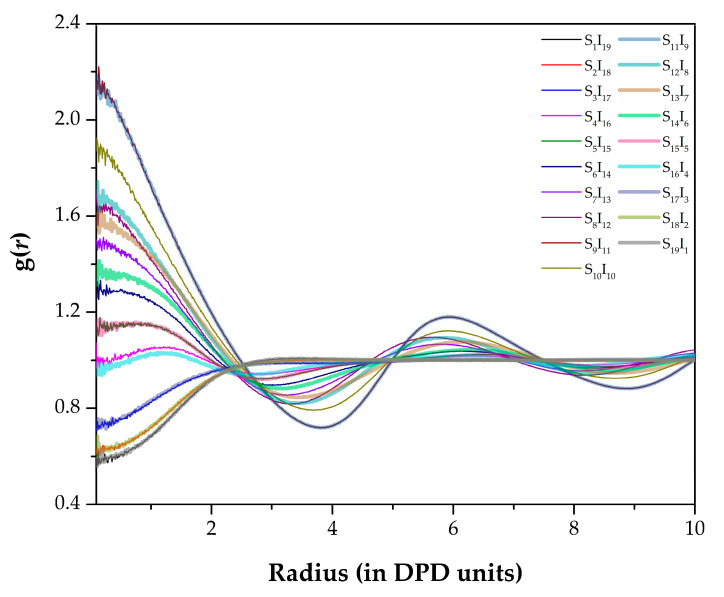
RDFs g(*r*) between chain–chain types in different coarse-grained models. *r* is plotted in the reduced DPD length unit. Some lines are bolded and transparent to ensure that all data can be seen.

Using an interchain RDF analysis, the morphologies both with similar or different physical arrangements were obviously noticed, as shown in Figure 2. The overlapping graphs indicated that the arrangements of coarse-grained beads were very similar and, vice versa, that the morphologies were different. The criteria to distinguish the similarity of the morphologies were a magnitude of g(*r*) and a graph shape.

**Figure 3:**
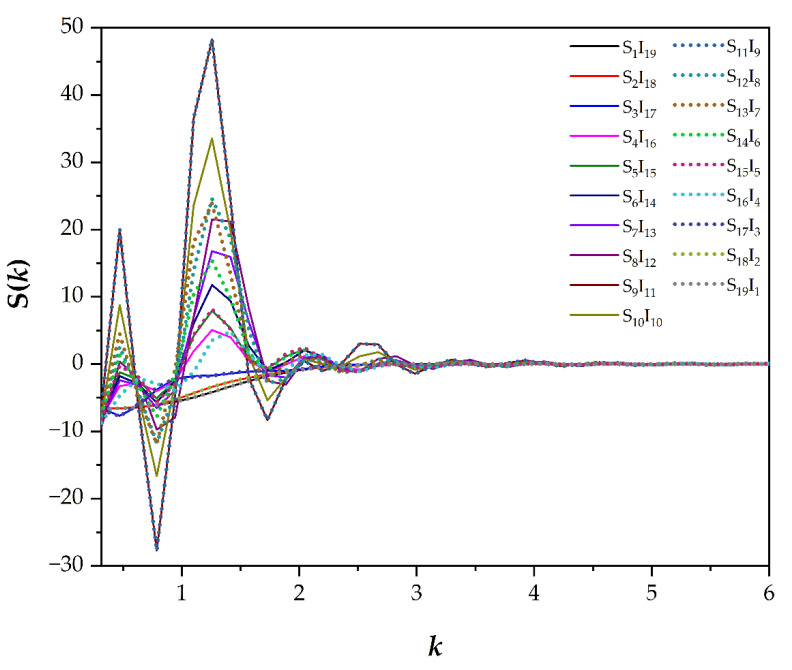
Structure factors S(*k*) in different coarse-grained models versus a frequency *k*. Some lines are illustrated in a dotted style to ensure that all data can be seen.

The structure factor is an experimentally observable parameter detected via a scientific instrument, such as the X-ray diffraction technique. This parameter depends on an arrangement of molecular structures in a material of interest and it is a useful method to distinguish the similarity or dissimilarity of materials, as has been reported in various papers [7,9,27,28]. S(*k*) can be understood in a similar way as the explanation of the RDF graph above. It was successfully used to classify the types of simulated morphologies, as illustrated in Figure 3. However, by solely using RDFs and S(*k*), one cannot group each morphology into the three main categories (isotropic, hexagonal and lamellar mesophases), as suggested by the order parameter. Therefore, a PCA analysis was carried out in this study and is discussed in the last section.

**Figure 4:**
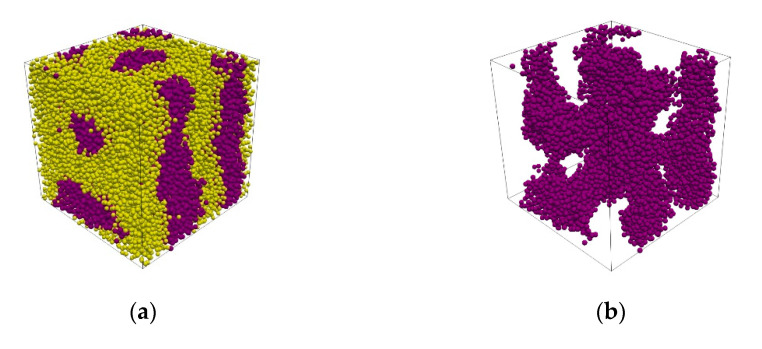

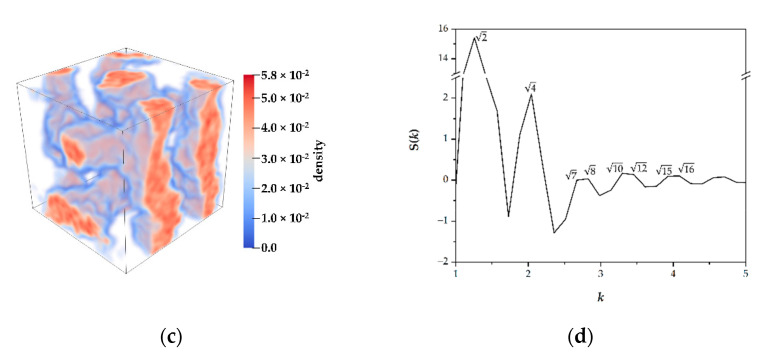
The coarse-grained model obtained from an S_14_I_6_: (**a**) an arrangement of the styrene (yellow) and isoprene (violet); (**b**) isoprene beads resulting solely in a skeleton of gyroidal morphology; and (**c**) a gradient of styrene density. The red regions correspond to high densities of isoprene and vice versa for the blue region. (**d**) The simulated S(*k*).

In this study, the S(*k*) of a simulated gyroidal morphology corresponded well to the experimentally reported data of the results detected by the X-ray diffraction technique. The peak position ratio of the experimentally observable refractions contained $\sqrt{3}$, $\sqrt{4}$, $\sqrt{7}$, $\sqrt{8}$, $\sqrt{10}$, $\sqrt{11}$, $\sqrt{12}$, $\sqrt{13}$, $\sqrt{15}$ and $\sqrt{16}$ [27]. The peaks appeared in our study as shown in Figure 4 and consisted of $\sqrt{2}$, $\sqrt{4}$, $\sqrt{7}$, $\sqrt{8}$, $\sqrt{10}$, $\sqrt{12}$, $\sqrt{15}$ and $\sqrt{16}$. The first peak slightly shifted to $\sqrt{2}$ and the peaks at $\sqrt{11}$ and $\sqrt{13}$ were missing from the simulated S(*k*); these observations could be a distortion of the gyroid. For example, two gyroidal morphologies obtained from S_6_I_14_ and S_14_I_6_ gave slight differences in the simulated S(*k*) due to the different structures, as shown in Figure 4 and Figure S1, respectively.

### 3.2. Principal Component Analysis (PCA) for Classification of Morphologies

**Figure 5:**
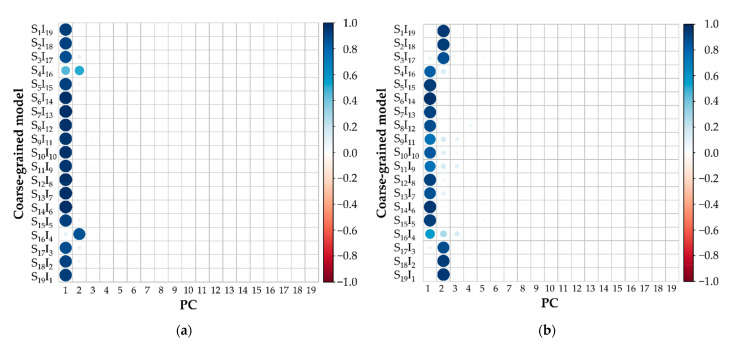
Correlation plots of (**a**) RDFs and (**b**) S(*k*) show a relationship between the variables of coarse-grained models and each principal component (PC). The diameter of each circle indicates the contribution of each variable in each PC. The color shades indicate the quality of each variable in each PC and the range of the quality is represented as a color gradient at the right-hand side.

Using the built-in packages, all classifications in the RDF and S(*k*) datasets were compressed into the first two components with a cumulative variance of 98.17% and 94.59%, respectively, as shown in Table 1. The correlation plots of each dataset (Figure 5) show that most of the data was compressed into the first two components, which matches with the corresponding cumulative variance percentages. In the RDF dataset (Figure 5a), S_4_I_16_ contributed to the two components equally. S_16_I_4_ mostly contributed to PC2 while the rest of the classifications were mostly compressed into PC1. In the S(*k*) dataset (Figure 5b), the datasets of S_4_I_16_ to S_16_I_4_ were mostly compressed into PC1, while the rest contributed to PC2.

**Figure 6:**
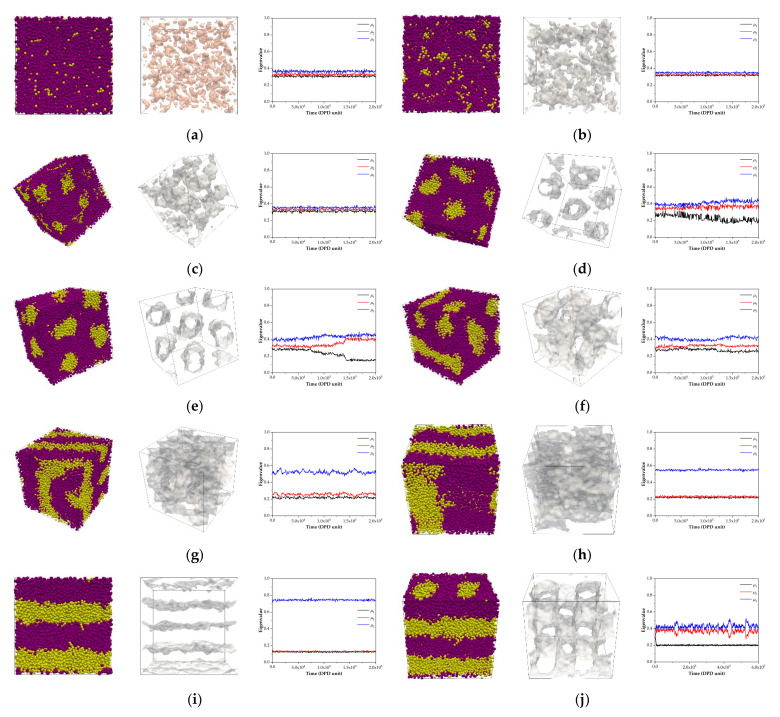
Panels in the composite figures illustrate apparent bead arrangements, isosurfaces between distinct bead types and the order-parameter sorting, from left to right, of (**a**) S_1_I_19_, (**b**) S_2_I_18_, (**c**) S_3_I_17_, (**d**) S_4_I_16_, (**e**) S_5_I_15_, (**f**) S_6_I_14_, (**g**) S_7_I_13_, (**h**) S_8_I_12_, (**i**) S_9_I_11_ and (**j**) S_10_I_10_, respectively. The order parameters were calculated using an executable, namely isosuefaces.exe, an implemented tool in the DL_MESO software. Yellow and violet beads represent coarse-grained beads of styrene and isoprene, respectively.

According to the DPD simulations, aggregations were only affected by their different ratios with the monomeric units of each block. To classify the obtained morphologies, we investigated them using three routes: (1) the apparent aggregation of coarse-grained beads, which is illustrated on the left-hand side of each sub-figure in Figure 6; (2) an isosurface investigation, which is shown in the middle of the sub-figure in Figure 6; and (3) a consideration of order parameters calculated using an embedded tool in the DL_MESO package, which is illustrated on the right-hand side of each sub-figure in Figure 6. Route 1 is a typical way to visualize and classify an obtained morphology due to its simplicity, with no need for post-processing. The structure can also be directly visualized using the trajectory from a simulation. However, the drawbacks of the long-length scale, resulting from its simulation box, may cause difficulties in morphological identification.

For isosurface and order parameter assessments, further calculations had to be carried out using the program isosurfaces.exe. With this tool, the volume of particle was smeared using a Gaussian function with a standard deviation σ:

(4) $$f(r)=\frac{1}{{{(2\pi \sigma }^{2})}^{\frac{3}{2}}}\exp\left(-\frac{{\left|{r-r}_{i}\right|}^{2}}{{2\sigma }^{2}}\right)’\;$$

where $r_{i}$ is the position of particle *i*. All sampling points on a regular orthogonal grid within a distance of 3σ can be obtained with this smearing function. The formations related to the densities of the system are written as a readable file for visualizing an isosurface in different species, which is considered as route 2 in the above visualization process.

According to Equation (4), the visualization of isosurfaces between different coarse-grained beads is very useful. We can see the real structure of each aggregation deep inside the structure, as shown in the middle of each sub-figure in Figure 6. The distinct morphologies are disorder, discrete clusters, hexagonally packed cylinders, connected clusters, defected lamellae, lamellae and connected cylinders, which were obtained from S_1_I_19_, S_2_I_18_, S_3_I_17_, S_4_I_16_, S_5_I_15_, S_6_I_14_, S_7_I_13_, S_8_I_12_, S_9_I_11_ and S_10_I_10_, respectively. The rich styrene models, S_11_I_9_, S_12_I_8_, S_13_I_7_, S_14_I_6_, S_15_I_5_, S_16_I_4_, S_17_I_3_, S_18_I_2_ and S_19_I_1_, exhibited a similar morphology as the rich isoprene models, as shown in Figure S2. All the structures observed using a combination of the three routes for classification are listed in Table 2.

**Figure 7:**
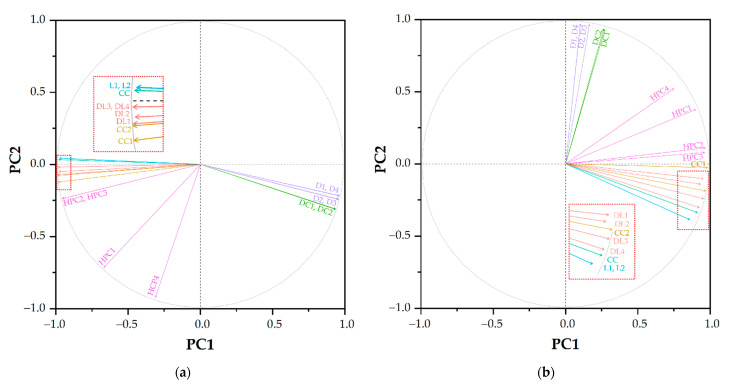
Loading plots of PCA analysis using (**a**) RDFs and (**b**) S(*k*) data. The colors violet, green, pink, yellow, red, blue and cyan correspond to disorder, discrete clusters, connected clusters, hexagonally packed cylinders, defected lamellae, lamellae and connected cylinders, respectively. The abbreviations are shown in Table 2.

The loading plots the morphologies (Figure 7) showed that each variable was grouped according to its morphology. Both lamellae (L1 and L2) classifications were almost identical to each other on both plots; hence, the eigenvector of the two morphologies was overlapping. The identical classification could also be observed for discrete clusters (DC1–DC2) and disorder (D1–D4, and D2–D3). The hexagonal packet cylinders (HPC1–4) cluster was also clustered in a distinct group in both plots. However, connected cylinders (CC) were presented differently in each plot. While CC was more similar to L1 and L2 in the RDF dataset (Figure 7a), the similarity of CC shifted to defected lamellae (DL) 4 in the S(*k*) dataset (Figure 7b). Furthermore, DL1–4 could be distinctively grouped in the RDFs dataset, but DL was clustered with connected clusters (CC1 and CC2).

## 4. Conclusions

Spatial intermolecular arrangement information derived from meso-scale simulations based on parameters from atomistic simulations were analyzed in dissipative particle dynamic (DPD) simulations of PS-*b*-PI diblock copolymers. In total, 19 coarse-grained models with different compositions were explored. The radial distribution function (RDF) and its Fourier-space counterpart, or structure factor (S(*k*)), were proposed using PCA as key characteristics for morphological identification and classification. The RDF and S(*k*) datasets were compressed into the first two components with a cumulative variance of 98.17% and 94.59%, respectively. The eigenvalues of the second moment of the tensor built up from the normal vectors on the isosurface of the density function for each particle in the DPD simulation were used as a guideline in the phase determination. To complete this analysis when the classification of some morphologies was limited, i.e., connected clusters and connected cylinders, an add-on analysis using PCA was effectively applied in this system. By employing PCA to reduce the dimensionality of the RDF and its Fourier-space counterpart from DPD simulations, a complicated arrangement of morphologies found in PS-*b*-PI copolymers could be successfully differentiated. Disorder, discrete clusters, hexagonally packed cylinders, connected clusters, defected lamellae, lamellae and connected cylinders were effectively grouped.

## Figures and Tables

**Table 1:** Eigenvalue and accumulated contribution ratio for the principal components (PCs) using RDF and S(*k*) data.

PC No.	RDFs			S(*k*)		
	Eigenvalue	Variance Percent(%)	Accumulated Variance Percent (%)	Eigenvalue	Variance Percent (%)	Accumulated Variance Percent (%)
1	1.67 × 10^1^	87.94	87.94	1.13 × 10^1^	59.69	59.69
2	1.94	10.23	98.17	6.63	34.89	94.59
3	2.15 × 10^−1^	1.13	99.30	6.27 × 10^−1^	3.30	97.88
4	6.93 × 10^−2^	0.36	99.67	2.14 × 10^−1^	1.13	99.01
5	3.05 × 10^−2^	0.16	99.83	1.61 × 10^−1^	0.85	99.86
6	1.52 × 10^−2^	0.08	99.91	2.36 × 10^−2^	0.12	99.99
7	6.31 × 10^−3^	0.03	99.94	1.90 × 10^−3^	0.01	100.00
8	2.71 × 10^−3^	0.01	99.95	5.77 × 10^−4^	0.00	100.00
9	2.33 × 10^−3^	0.01	99.97	2.66 × 10^−4^	0.00	100.00
10	2.15 × 10^−3^	0.01	99.98	2.55 × 10^−5^	0.00	100.00
11	1.13 × 10^−3^	0.01	99.98	6.06 × 10^−6^	0.00	100.00
12	8.03 × 10^−4^	0.00	99.99	4.77 × 10^−6^	0.00	100.00
13	6.66 × 10^−4^	0.00	99.99	1.28 × 10^−6^	0.00	100.00
14	4.80 × 10^−4^	0.00	99.99	8.82 × 10^−7^	0.00	100.00
15	3.11 × 10^−4^	0.00	100.00	5.52 × 10^−7^	0.00	100.00
16	2.92 × 10^−4^	0.00	100.00	4.21 × 10^−7^	0.00	100.00
17	2.39 × 10^−4^	0.00	100.00	2.44 × 10^−7^	0.00	100.00
18	1.90 × 10^−4^	0.00	100.00	2.05 × 10^−7^	0.00	100.00
19	1.26 × 10^−4^	0.00	100.00	2.64 × 10^−8^	0.00	100.00

**Table 2:** Coarse-grained models and their morphological classification.

Volume Fraction of Styrene	Coarse-Grained Model	Morphology (Its Abbreviation)
0.05	S_1_I_19_	Disorder (D1)
0.10	S_2_I_18_	Disorder (D2)
0.15	S_3_I_17_	Discrete clusters (DC1)
0.20	S_4_I_16_	Hexagonally packed cylinders (HPC1)
0.25	S_5_I_15_	Hexagonally packed cylinders (HPC2)
0.30	S_6_I_14_	Connected clusters (CC1)
0.35	S_7_I_13_	Defected lamellae (DL1)
0.40	S_8_I_12_	Defected lamellae (DL2)
0.45	S_9_I_11_	Lamellae (L1)
0.50	S_10_I_10_	Connected cylinders (CC)
0.55	S_11_I_9_	Lamellae (L2)
0.60	S_12_I_8_	Defected lamellae (DL3)
0.65	S_13_I_7_	Defected lamellae (DL4)
0.70	S_14_I_6_	Connected clusters (CC2)
0.75	S_15_I_5_	Hexagonally packed cylinders (HPC3)
0.80	S_16_I_4_	Hexagonally packed cylinders (HPC4)
0.85	S_17_I_3_	Discrete clusters (DC2)
0.90	S_18_I_2_	Disorder (D3)
0.95	S_19_I_1_	Disorder (D4)

## Data Availability

Not applicable.

## Supplementary Materials

The following are available online at https://www.mdpi.com/article/10.3390/polym13162581/s1, Figure S1: Structure factors S(*k*) between chain–chain types of the gyroidal morphologies of S_6_I_14_ and S_14_I_6_; Figure S2: Panels in the composite figures illustrate apparent bead arrangements, isosurfaces between distinct bead types and order-parameter sorting, from left to right, of (**a**) S_11_I_9_, (**b**) S_12_I_8_, (**c**) S_13_I_7_, (**d**) S_14_I_6_, (**e**) S_15_I_5_, (**f**) S_16_I_4_, (**g**) S_17_I_3_, (**h**) S_18_I_2_ and (**i**) S_19_I_1_, respectively. The order parameters were calculated using an executable, namely isosueface.exe, that is an implemented tool in DL_MESO software. Yellow and violet beads represent coarse-grained beads of styrene and isoprene, respectively, Table S1: Parameters used in DPD simulation in dimensionless unit, Script S1: Structural knowledge analysis and PCA visualization.
