# Supplementary material for: Characteristic Structural Knowledge for Morphological Identification and Classification in Meso-Scale Simulations Using Principal Component Analysis
Source: Polymers (Basel). 2021 Aug 4;13(16):2581. doi: 10.3390/polym13162581 (PMC8400772; doi:10.3390/polym13162581)
Supplement: Supplementary file 1 [file polymers-13-02581-s001.zip › polymers-1300875-supplementary.pdf]

Supplement Materials

Characteristic structural knowledge for morphological identification and classification in meso-scale simulations using principal component analysis

Natthiti Chiangraeng [1], Michael Armstrong [1], Kiattikhun Manokruang [1], Vannajan Sanghiran Lee [2,3], Supat Jiranusornkul [4], and Piyarat Nimmanpipug [1,3,*]

[1] Department of Chemistry, Faculty of Science, Chiang Mai University, Chiang Mai, 50200, Thailand; natthiti.c@gmail.com (N.C.); armstrongmichael119@gmail.com (M.A.); kiattikhun.m@cmu.ac.th (K.M.)
[2] Department of Chemistry, Faculty of Science, University of Malaya, 50603 Kuala Lumpur, Malaysia; vannajan@um.edu.my (V.S.L.)
[3] Center of Excellence for Innovation in Analytical Science and Technology (I-ANALY-S-T), Chiang Mai University, Chiang Mai 50200, Thailand
[4] Department of Pharmaceutical Sciences, Chiang Mai University, Chiang Mai 50200, Thailand; supat.jira@cmu.ac.th (S.J.)
* Correspondence: piyarat.n@cmu.ac.th (P.N.)

Table S1. Parameters used in DPD simulation in dimensionless unit.

**Parameter**	
Particle number, N	24,000
Simulation box volume	8000
Coarse-grained degree, N_m	5
Spring constant, C_{ij}	4
Thermal energy, k_BT	1
Repulsion parameter of the same beads, a_{ii}	131.50
Repulsion parameter of the different beads, a_{ij} at temperature of 393 K	142.18
Time step, Δt	0.01
Equilibrium step number	1×10^6
Production step number	$\geq 1 \times 10^7$

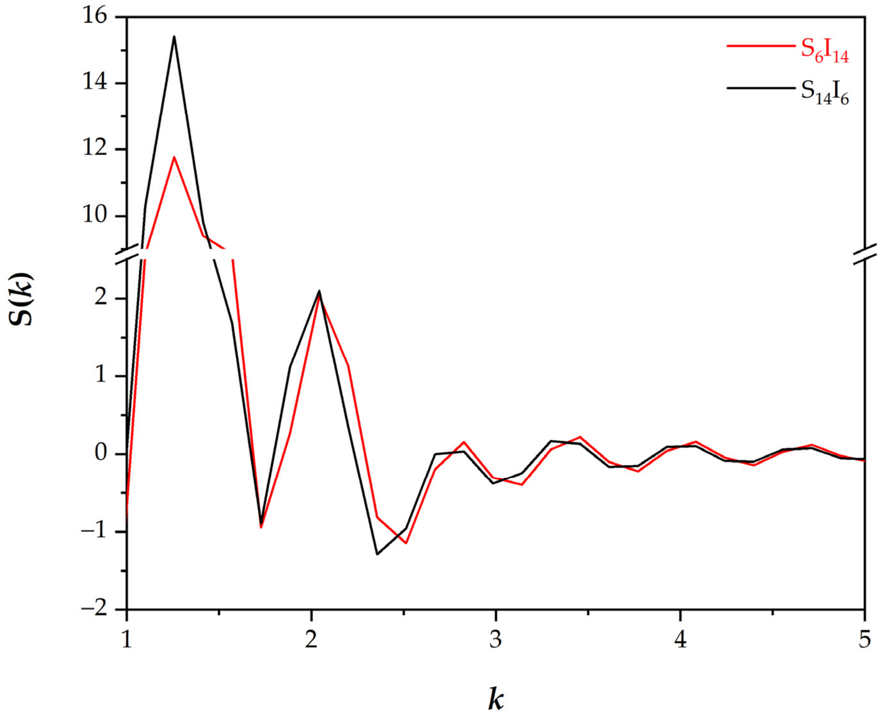

Figure S1. Structure factors S(*k*) between chain-chain types of the gyroidal morphologies of S_6I_{14} and $S_{14}I_6$ were represented as red and black lines, respectively.

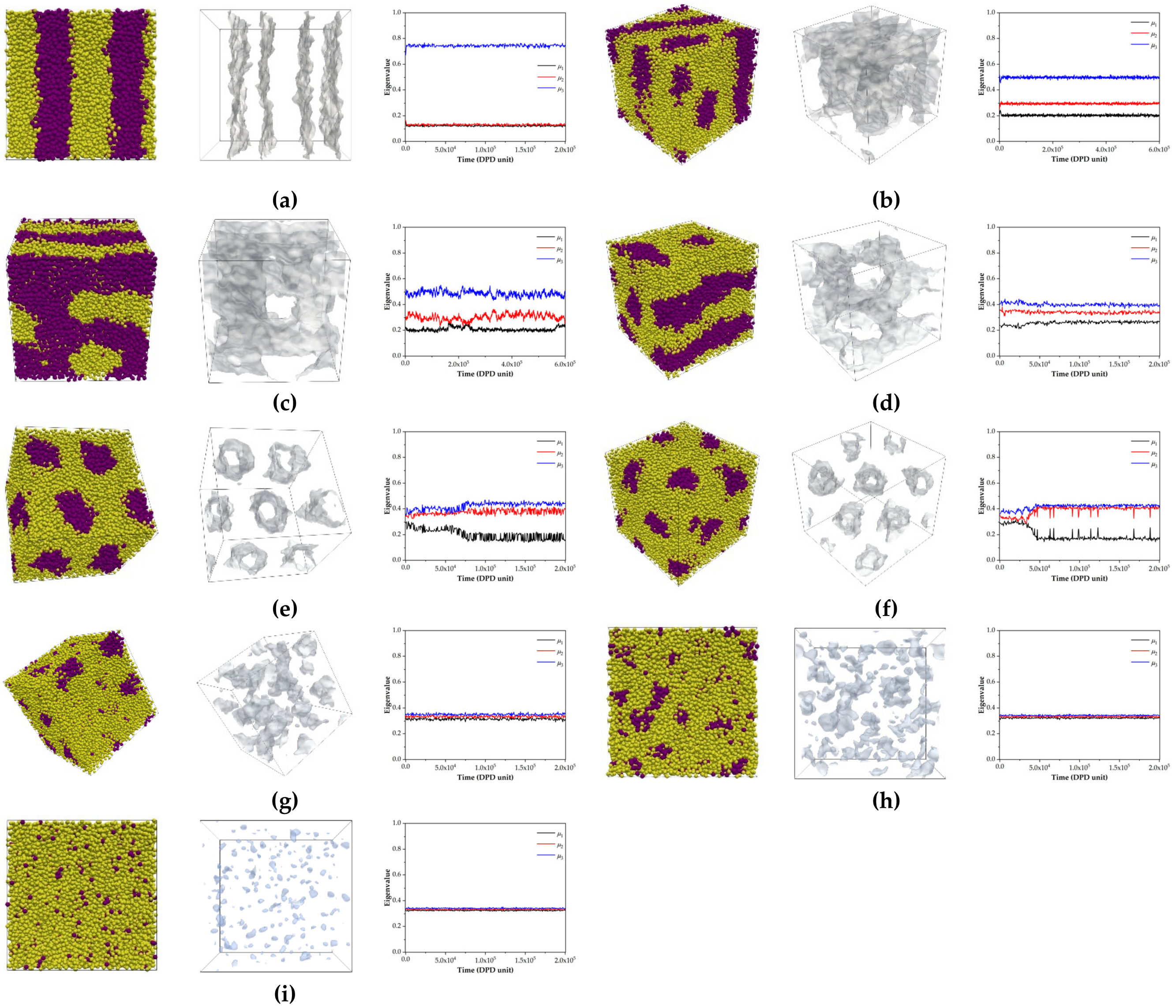

Figure S2. Panels in the composite figures illustrate apparent bead-arrangement, isosurface between distinct bead types and order parameter sorting from the left to right of (a) $S_{11}I_9$, (b) $S_{12}I_8$, (c) $S_{13}I_7$, (d) $S_{14}I_6$, (e) $S_{15}I_5$, (f) $S_{16}I_4$, (g) $S_{17}I_3$, (h) $S_{18}I_2$, and (i) $S_{19}I_1$, respectively. The order parameters were calculated using an executable, namely isosueface.exe, that is an implemented tool in DL_MESO software. Yellow and violet beads represent coarse-grained beads of styrene and isoprene, respectively.

Script S1: Structural knowledge analysis and PCA visualization
7/29/2021

Libraries
```
library(tidyverse)
library(ggforce)
library(factoextra)
library(ggrepel)
library(corrplot)
```

Data
```
data <- <name_of_excel_file>[<range_of_row>,<range_of_column>]
data
data.frame <- as.matrix.data.frame(data)
data.frame
```

Run the PCA
```
pca <- prcomp(data.frame,scale = TRUE)
pca

summary(pca)


eig.val <- get_eigenvalue(pca)
eig.val


options(ggrepel.max.overlaps = Inf)
fviz_eig(pca)
fviz_pca_ind(pca,title = " ",geom = "point",col.ind = "cos2", gradient.cols = c("#00AFBB", "#E7B800", "#FC4E07"))

fviz_pca_var(pca,title = " ",label = "none",col.var = "cos2",gradient.cols = c("#00AFBB", "#E7B800", "#FC4E07"))

res.var <- get_pca_var(pca)
res.var$coord
res.var$contrib
res.var$cos2


corrplot(res.var$cos2, is.corr = TRUE,)
```
#
